# Supplementary material for: HLA-DPB1 Reactive T Cell Receptors for Adoptive Immunotherapy in Allogeneic Stem Cell Transplantation
Source: Cells. 2020 May 20;9(5):1264. doi: 10.3390/cells9051264 (PMC7290340; doi:10.3390/cells9051264)
Supplement: Supplementary file 1 [file cells-09-01264-s001.pdf]

Figure S1

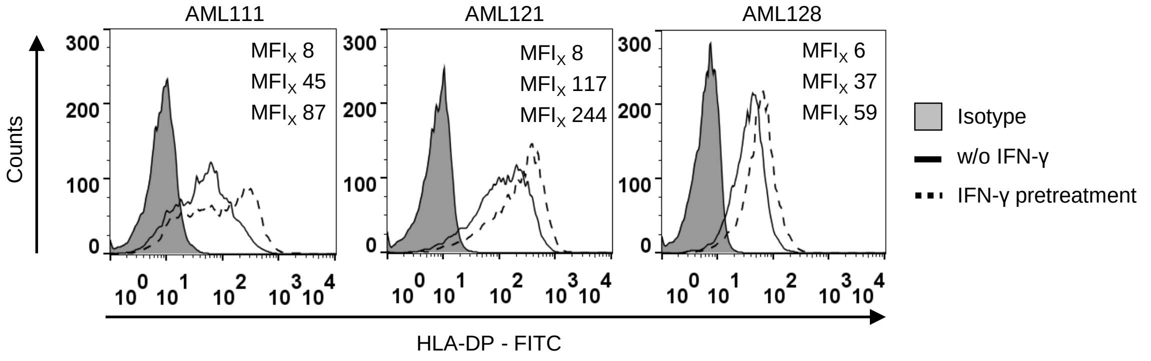

Supplementary Figure 1. HLA-DP surface expression on primary AML blasts. AML samples from individual patients (AML111, AML121, AML128) were analyzed cytofluorometrically for surface expression of HLA-DP. Cells were either left untreated (solid black line) or pretreated with IFN-γ (500 IU/mL) for 24 h (black dotted line) before staining with HLA-DP specific mAb. Isotype mAb stained cells served as control (filled black line). MFI values are shown in the following order: Isotype (upper value), untreated (middle value), IFN-γ pretreated (lower value).

Figure S2

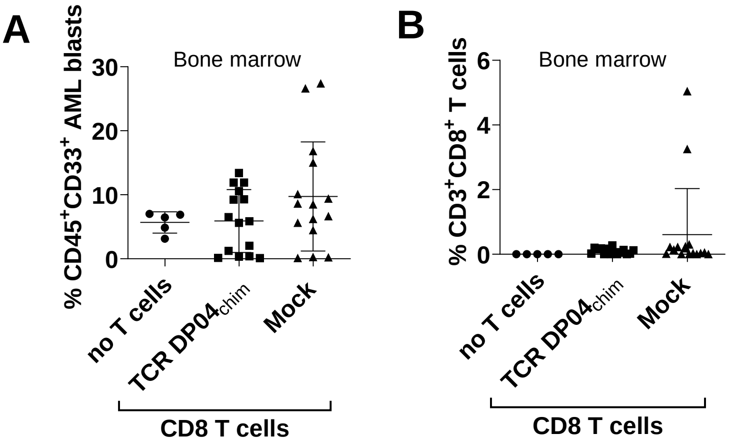

Supplementary Figure 2. Anti-leukemic activity of TCR $DP04_{chim}$ modified CD8 T cells in AML engrafted NSG mice. Sublethally irradiated (1.5 Gy) NSG mice were intravenously injected with 4 x 10^6 primary blasts of AML167 (HLA-DPB1*04:01/17:01). On day 21, when leukemia engraftment reached a level of 1-5 % in bone marrow as confirmed in identically treated control mice, 1 x 10^7 CD8 T cells retrovirally transduced with TCR $DP04_{chim}$ (n=15) or empty vector(Mock; n=15) were intravenously injected along with single doses of rh IL-2 (1000 U) and FcIL-7 (20 µg). Animals in the control group without T cells (n=5) only received rh IL-2 and Fc-IL-7. On day 28 (i.e. 7 days after T cell transfer), **(A)** AML burden ($CD45^+CD33^+$ cells) and (**B**) T cell frequencies ($CD3^+CD8^+$ cells) were analyzed in bone marrow. Results are pooled from three independent experiments. Symbols represent individual mice and horizontal bars mark mean values with standard deviation.

Figure S3

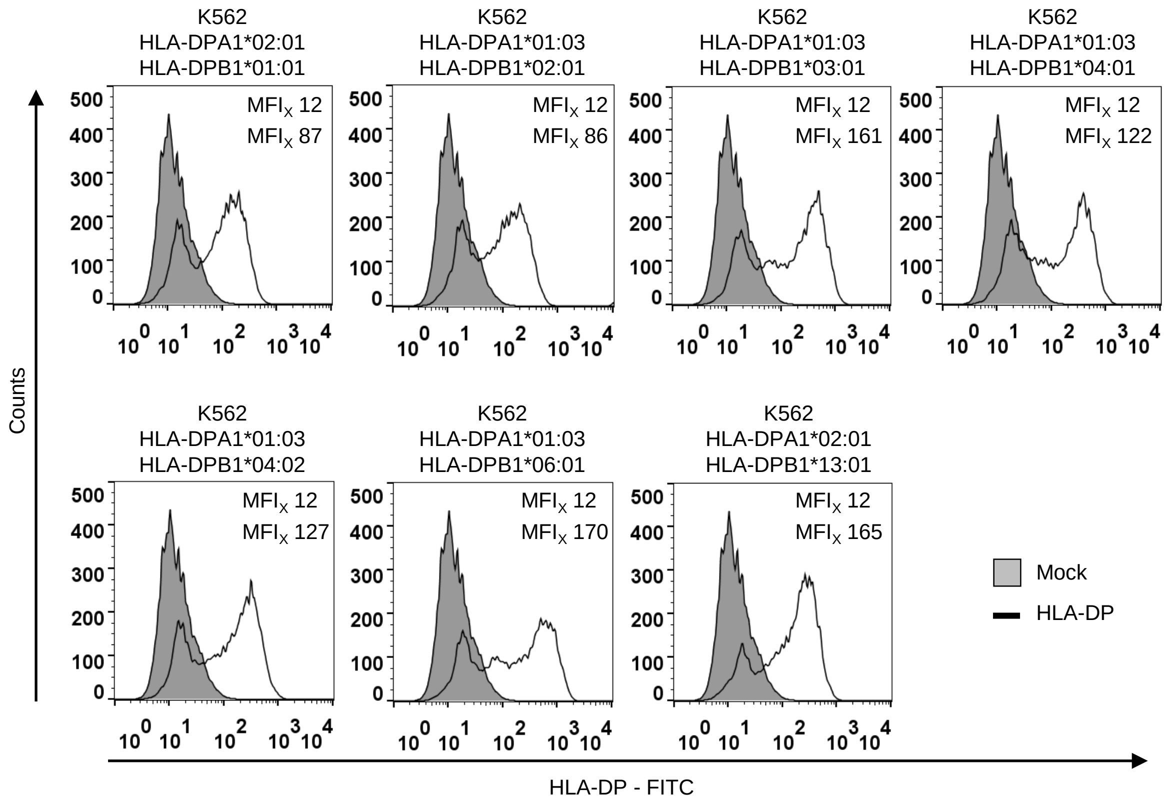

Supplementary Figure 3. HLA-DP cell surface expression on K562 cells. K562 cells were analyzed for surface expression of HLA-DP by flow cytometry 16-20 h after RNA transfection. Cells were either electroporated with IVT-RNA (10 µg each) encoding for the indicated HLA-DPA1* and -DPB1* alleles (solid black line) or electroporated without RNA (Mock, filled grey line). MFI values for HLA-DP surface staining are shown in the following order: Mock (upper value), HLA-DP electroporated (lower value).

Figure S4

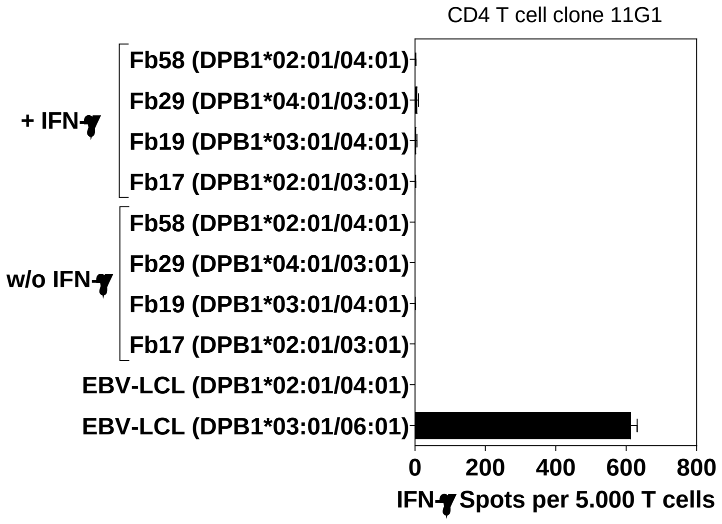

Supplementary Figure 4. Reactivity of clonal CD4 T cells 11G1 against primary fibroblasts. T cells from clonal CD4 population 11G1 were incubated with untreated or IFN-γ pretreated primary fibroblasts from individual HLA-DPB1*03:01 positive and negative donors at an E:T ratio of 0.1:1. IFN-γ spot production was measured after 16-20 h. EBV-LCL from HLA-DPB1*03:01 positive and negative donors served as controls. Standard deviation of mean of two technical replicates is shown.

Figure S5

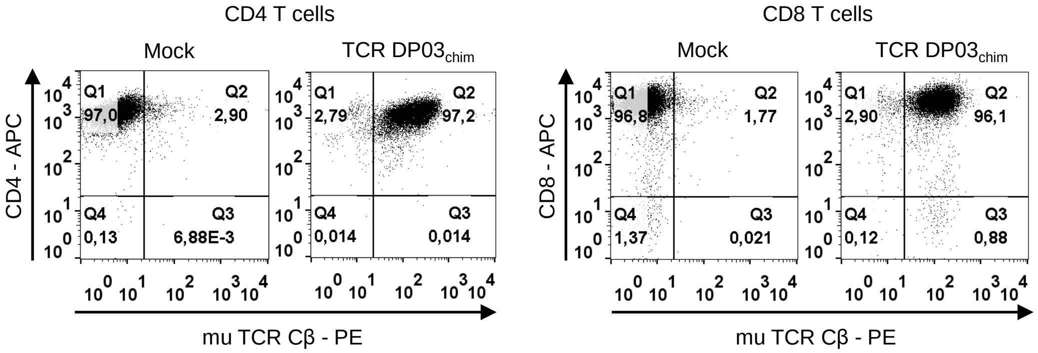

Supplementary Figure 5. TCR expression of TCR $DP03_{chim}$ redirected T cells. Immunomagnetically selected and prestimulated human CD4 (left panels) and CD8 T cells (right panels) from an HLA-DPB1*03:01 negative healthy donor were transfected with TCR $DP03_{chim}$ coding RNA (CD4 TCR $DP03_{chim}$ and CD8 TCR $DP03_{chim}$) or without RNA (CD4 Mock and CD8 Mock) and analyzed after 16-20 h by flow cytometry for expression of CD4, CD8, as well as of TCR $DP03_{chim}$ using murine TCR constant β domain (mu TCR Cβ)-specific mAb.

Figure S6

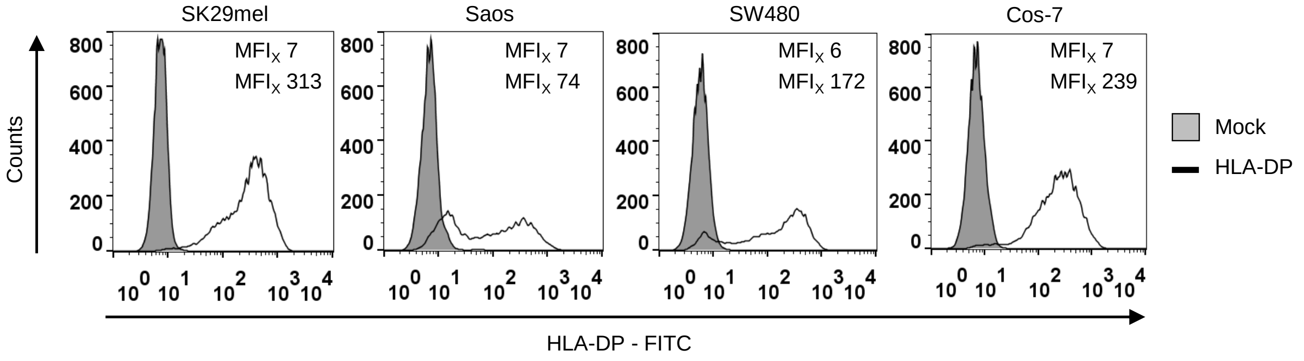

Supplementary Figure 6. HLA-DP cell surface expression on tumor cell lines. Indicated human (SK29mel, Saos, SW480) and primate (Cos-7) tumor cell lines were analyzed cytofluorometrically for surface expression of HLA-DP 16-20 h after RNA transfection. Cells were either electroporated with IVT-RNA (10 µg each) encoding for HLA-DPA1*01:03 and -DPB1*04:01 alleles (solid black line) or electroporated without RNA (Mock, filled grey line). MFI values for HLA-DP surface staining are shown in the following order: Mock (upper value), HLA-DP electroporated (lower value).
